# The Attitude of Great Writers towards Disease

**Published:** 1892-08-27

**Authors:** 


### The Attitude of Great Writers Towards Disease.

### XI.—THE LITERATURE OF DESPAIR.

Almost every one is attacked at some period of his life by a subtle tendency, akin to disease, towards despair in the possibilities of the future and revolt against the realities of the present. The tendency can be traced back as far as men's first recorded utterances about themselves, and finds expression in the literature of every age and every nation. On another occasion we may examine some of the records of blighted lives which constitute such cheerful reading in our own *fin de siècle.* At the present moment we wish to recall three romances which appeared about the close of last century in Germany, Italy, and France, holding up to admiration and sympathy certain conditions of mind and body under which hope is altogether extinguished, and life, becoming unendurable, is rejected. For it was these romances which glorified a habit of mind not before regarded with great favour by writers of consideration. They give perhaps more insight into the spirit of their age than many works of sounder purpose, and they stirred a wave of emotion among the young men of the day which touched our own shores too, and roused an eager response among the poets and thinkers of England.

The form of each romance is simplicity itself, and was determined by Goethe, who was first in the field. Written in the person of a very young man, each recounts the tragedy of an ardent and sensitive nature, blighted in affection and blighted in career. Something of autobiography was thrown by each author into his romance. Indeed, Goethe in later life acknowledged to Bettina that he had written the sorrows of Werther in order to rid himself of the tendency towards suicide which haunted him perpetually. Werther's story has been so charmingly summarised by Thackeray that at the risk of disrespect to the original his version is here inserted for the benefit of those to whom that is unknown.

> Werther had a love for Charlotte
> Such as words could never utter;
> Would you know how first he met her?
> She was cutting bread and butter.
> Charlotte was a married lady,
> And a moral man was Werther,
> And for all the wealth of Indies,
> Would do nothing for to hurt her.
> So he sighed, and pined, and ogled,
> And his passion boiled and bubbled,
> Till he blew his silly brains out,
> And no more was by it troubled.
> Charlotte having seen his body,
> Borne before her on a shutter,
> Like a well-conducted person
> Went on cutting bread and butter.

The reader who wishes to have any idea of the exquisite poetic colouring of the story would do well not to be contented with this version. The sorrows of Werther is essentially a drama of sensation, a development of the doctrines of Rousseau. As such nothing could be more finely conceived than this tracing of the successive blows which loosen the hold upon life of a highly-strung nature, accustomed to exaggerate every emotion, and absolutely untrained in self-restraint and endurance. That it exercised a very bad influence on the youth of the generation is indisputable. Except at the very last, when his egoism and bluntness of perception towards others reach a point of acute brain sickness, Werther is always a sympathetic creation, He became, unhappily, to many discontented youths, a justification for self-destruction.

Ugo Foscolo, who published his "Jacopo Ortis" in 1802, was indebted to Goethe for much of his story, and, indeed, copied him with scrupulous but probably unconscious fidelity in almost every detail. But Jacopo Ortis is a very different person from Werther, and the Italian romance has many elements wanting in the German. Proscribed from Venice after the Treaty of Campo Formio, which sold his native land to the Austrians, Ortis takes refuge among the Enganean hills. Here in exile he encounters Teresa, who, like Werther's Lottchen, is, in the language of Mr. Augustus Moddle, about "to become Another's." But the hopeless love of Ortis for Teresa, though the ultimate cause of his suicide, is not the leading motive in the story. It is expatriation, shame for his fellow-citizens, despair for his country, all the complex bitterness of an exiled life which lie at the root of his despair, and make the life which flickered into sudden animation under Teresa's charm, sink down and expire at the final separation. It was this element in the book written in the heart's blood of the author, which made it the exponent of young Italy's conscious shame and repression. It is this which accounts for the enormous popularity of a romance containing very much that is sickly and contrary to good taste. The despair of Ortis was the despair of a nation; its expression was a national relief, as the groan of a stupified sufferer awaking to consciousness.

Thus, in Germany and Italy all the outward and inward marks of what might be called a diseased estimate of self became the fashion. It was a superior thing to affect a morbid seclusion; it was the mark of a higher order of mind to exaggerate disappointment into despair, and to mistake indolent indecision for purity of aim.

In France the same ideas found expression in a writer like Goethe, eminently sane in other respects. Chateaubriand's René is not a very pleasing person. He is a young man of means and leisure, early left an orphan with an only sister. He lives a life of seclusion on his estates until, for want of occupation, he falls into a condition of deadly boredom. The boredom resolves itself into a kind of Weltschmerz, the possession of which lends him a certain dignity among his surroundings. He is assured "it is no matter of surprise if he suffers more from life than another; a great soul must be capable of more grief than a little one." Here is the plague spot from which the whole mischief starts. It is exactly this slighting of the sorrows of other men, from which springs the diseased conception of man's claim upon the world for happiness, and the querulous self-importance which magnifies his own share of the common burden. For there is no standard by which we can measure our griefs with those of a fellow sufferer; nor can we dare, in the mystery which hides our souls one from another, to decree that he bears less who bears better.

René, being deserted by his sister, resolves to travel, and passes through Europe, "seeking only an unknown good, the instinct of which pursued" him. Needless to say, the "unknown good" was not to be found in picture-galleries or ruins, nor were the acquaintances he formed of a nature to lend fresh inspiration to one already glorying in having tasted and rejected all the best which life could offer. He returns home confirmed in the isolation which he deems the superiority of his nature imposes upon him. There is no need to dwell upon the peculiarly French catastrophe which reduced René to the final point of despair. It is matter for thankfulness that he stops short at self-destruction, and confines himself to perpetual exile among the Natchez Indians in Louisiana. His reasons for this act of self-restraint are characteristic: "I had no desire to die after I became really unhappy. My sorrow had become an occupation which filled all my moments." Positively he had now something to do, and his reason was saved.

It seems from these three studies, written by men who had lived through corresponding phases of thought, that this form of pain, which daunts and paralyses the mind like some hideous spiritual disease, is a pain which chiefly attacks those removed for any reason from actual contact with human suffering in its most real aspects. It lurks in the study and the drawing-room, and seizes its victims among the dreamers and the unemployed. Among the noble band of sisters who toil and wake round the beds of the sick in simple performance of duties, the bare thought of which would once have raised a shudder, are there not hundreds who have learnt to recognise this truth? In healing others has not the old restless self-torment been stilled? Has not the very disease you combat been as the lance of Achilles, holding at one hand death, and healing at the other; so that in God's wise purposes the problem has found solution at last, over which men fret and wrangle alone in ever-deepening despair.

"Pain is God's medicine to heal the soul."
So wrote the wise divine. And one who lay
Grievously tortured, quickly answered, "Nay,
It was the devil's hand which mixed the bowl—
Pain is but poison, oozing from the whole
Corrupted mass of sin and ignorance,
Wherein men grovel, slothful to advance,
Crying 'Thy will be done.'" Then softly stole
The answer: "Baleful herbs yield healing wine:
Doth not the great Physician mould at will
All jarring elements of mortal strife,
And use them for His purposes divine,
Evoking by the magic of His skill
From poison cure—from death eternal life?"

